# Roles of specialized metabolites in biological function and environmental adaptability of tea plant (*Camellia sinensis*) as a metabolite studying model

**DOI:** 10.1016/j.jare.2020.11.004

**Published:** 2020-11-09

**Authors:** Lanting Zeng, Xiaochen Zhou, Yinyin Liao, Ziyin Yang

**Affiliations:** a Guangdong Provincial Key Laboratory of Applied Botany & Key Laboratory of South China Agricultural Plant Molecular Analysis and Genetic Improvement, South China Botanical Garden, Chinese Academy of Sciences, No. 723 Xingke Road, Tianhe District, Guangzhou 510650, China; b Center of Economic Botany, Core Botanical Gardens, Chinese Academy of Sciences, No. 723 Xingke Road, Tianhe District, Guangzhou 510650, China; c University of Chinese Academy of Sciences, No.19A Yuquan Road, Beijing 100049, China

**Keywords:** *Camellia sinensis*, Tea, Basic biology, Specialized metabolite, Biological function, Environmental adaptability

## Abstract

**Background:**

Tea is the second most popular beverage globally after water and contains abundant specialized metabolites. These metabolites give tea unique quality and are beneficial to human health. Some secondary metabolites are produced to help plants, including tea plants (*Camellia sinensis*), adapt to variable environment and grow normally. Therefore, whether abundant specialized metabolites have biological functions and play roles in the environmental adaptability of tea plants is of interest.

**Aim of review:**

Research progress regarding the biological functions of specialized metabolites (including catechins, l-theanine, caffeine, and volatile compounds) in tea plants is summarized. Furthermore, the main and characteristic scientific questions regarding tea plant growth in contrast to other economic crops are proposed, including (i) how tea plants adapt to acid soils, (ii) why tea plants have fewer diseases, and (iii) why tea plants and tea green leafhoppers have a symbiotic relationship. Accordingly, the potential adaptive mechanism is summarized, which is related to the function of specialized metabolites in tea plants.

**Key scientific concepts of review:**

This is the most in-depth investigation of biological functions of volatile compounds in tea plants. Direct *in vivo* evidence in tea plants shows that volatile compounds help defend against insects through plant-to-plant signaling. Furthermore, abundant specialized metabolites are speculated to contribute to the environmental adaptability of tea plants. However, further *in vivo* evidence and exploration of relevant mechanisms are required for all aspects discussed. This review provides an important reference for basic biological research on the tea plant as a specialized metabolite studying model.

## Graphical abstract

**Figure:**
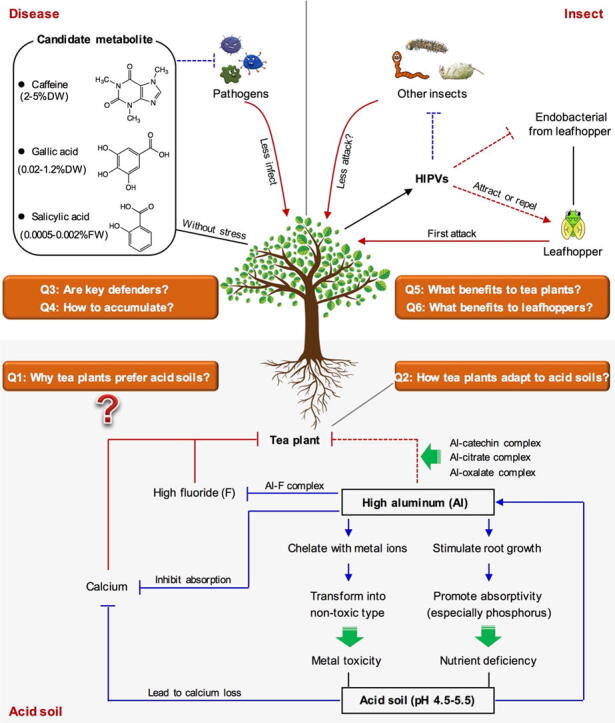

## Introduction

Tea plant (*Camellia sinensis*) belongs to genus *Camellia* L. and family Theaceae. A documentary has highlighted that the tea plant bears no sweet fruit, but plays a significant role in the economies of more than 60 countries and lives of three billion people (https://www.jianshu.com/p/c90ecf6ccb6b; https://www.iqiyi.com/v_19ru8mjinc.html). Compared with other plants, secondary metabolites of tea plants not only endow tea with unique quality, but also benefit human health. As an important economic plant, tea has been studied in many fields, including health, food production, and culture. Using “tea” or “*Camellia sinensis*” as search keywords, 57,080 articles were retrieved from the Web of Science database (core collection) from 1990 to 2020 (data searched on 24th March 2020) (http://apps.webofknowledge.com/WOS_GeneralSearch_input.do?product=WOS&SID=6DapT2MLzEJxnXdILOF&search_mode=GeneralSearch). The five most represented countries and the ten most represented research fields for these articles are shown in Table 1. Tea research conducted from a plant science perspective was found to be limited.

**Table 1:** Study on “tea” or “*Camellia sinensis*” from 1990 to 2020.

Aspect	Details	Number of articles	Percentage of total articles (%)
Country	America	11,514	20.2
	China	11,293	19.8
	Japan	6214	10.9
	India	3598	6.3
	England	2452	4.3
Research field	Chemistry	12,335	21.6
	Food Science Technology	9496	16.6
	Biochemistry Molecular Biology	6214	10.9
	Pharmacology Pharmacy	6092	10.7
	Nutrition Dietetics	4892	8.6
	Agriculture	4395	7.7
	Engineering	3087	5.4
	Plant Sciences	2555	4.5
	Oncology	2551	4.5
	Materials Science	2323	4.1

The data were searched on 24th March 2020 and obtained from the Web of Science database (core collection) (http://apps.webofknowledge.com/WOS_GeneralSearch_input.do?product=WOS&SID=6DapT2MLzEJxnXdILOF&search_mode=GeneralSearch). Only the five most represented countries and the ten most represented research fields for the articles are shown in the table.

However, in recent years, with the development of molecular biology methods, basic research on tea plant biology has become a popular topic in tea science field, with some progress made [1], [2], [3], [4], [5], [6]. Although information from other plants provides a good reference for tea plant studies, the starting point of the research should be carefully considered. We should establish why tea is chosen as a research object, and consider the characteristic scientific questions regarding tea plant growth or production in the tea industry. Furthermore, the rapid development of omics technology has greatly influenced basic research on tea plant biology [7], [8], [9], [10], [11]. Much research provides correlation analysis and confirmatory evidences are needed. In this review, we focus on the role of specialized metabolites in tea plants with the aim of answering characteristic scientific questions regarding tea plant growth.

From a human perspective, secondary metabolites act as important components for economic crop quality. Various secondary metabolites contribute to the quality and function of tea [12]. During tea plant growth (preharvest stage) and the tea manufacturing process (postharvest stage), many stresses are used to “modify” tea metabolites [1], [2], [13]. At the preharvest stage, shading treatment (abiotic stress) has been shown to enhance free amino acids and aromatic aroma compounds, and reduce catechins [14], [15], [16]. Furthermore, tea leaf attacked by tea green leafhoppers (biotic stress) increases honey-fruit aroma compounds [1], [17]. Compared with other economic crops, the postharvest manufacturing process is an important stage for improving tea quality, especially regarding tea aroma. A comprehensive investigation of aroma compound formation at this stage showed that jasmonic acid (JA)–key genes–characteristic aroma compounds was the main regulatory route [18], [19], [20], [21], [22]. To date, much research has been conducted to clarify the mechanism involved in improving tea quality with stresses.

From a plant perspective, many secondary metabolites play multiple biological functions in protecting plants against various stresses to adapt to the variable environments. To date, multiple biological functions of metabolites in many plant species, including tea plants, have been studied. In this review, important research progress on the biological functions of specialized metabolites is summarized. Furthermore, researchers have attempted to elucidate the mechanism of tea plant adaptability to the environment. The main and characteristic scientific questions regarding tea plant growth in contrast to other economic crops are also proposed. Accordingly, we summarize the potential adaptive mechanism, which relates to the roles of specialized metabolites in tea plants. This review provides an important reference for basic biological research on the tea plant as a specialized metabolite studying model.

## Biological function of specialized metabolites in tea plants

**Fig. 1:**
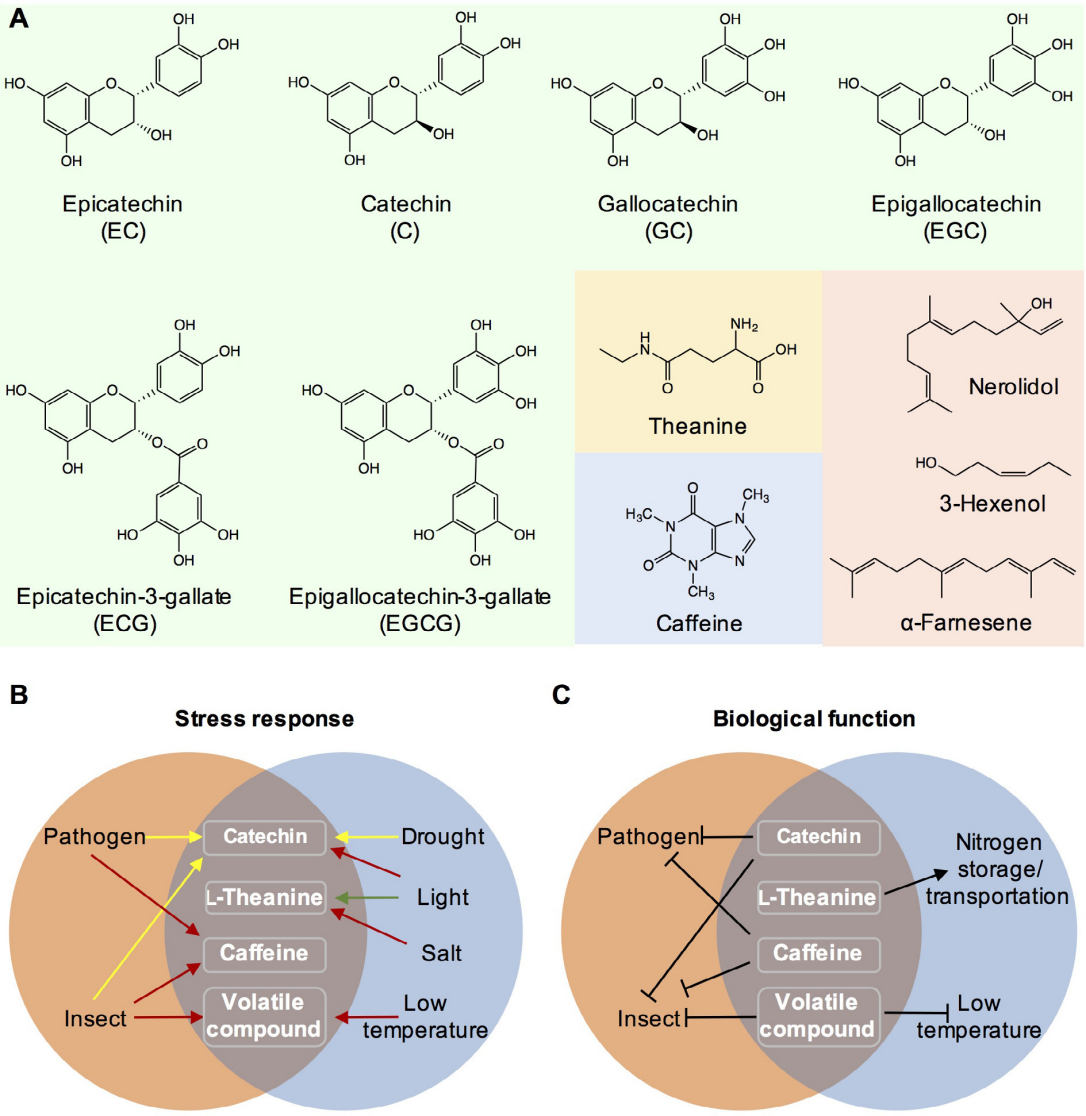
Specialized metabolites in tea plants and their potential biological functions. (A) The structures of specialized metabolites in tea plants cited in the review. The metabolites labelled in the green background are catechins, and the pink background are volatile compounds. (B) Stress response of specialized metabolites in tea plants. Red arrow indicates that the content of metabolite increased under stress. Green arrow indicates that the content of metabolite decreased under stress. Yellow arrow indicates that the content of metabolite showed different changing tendency under the infection of different pathogens or attack of different insects or drought treatment with different degrees. (C) Potential biological function of specialized metabolites in tea plants.

Plants contain numerous secondary metabolites, which are usually divided into three types, namely, terpenes (or terpenoids), phenolic compounds (such as flavonoids, isoflavonoids, anthocyanins, lignins, and tanins), and nitrogen-containing compounds (such as alkaloids, cyanogenic glycosides, glucosinolates, and nonprotein amino acids). The biological activity of these metabolites has been widely studied, with most reported to play important anti-pathogen and anti-insect roles [23]. Furthermore, terpenoids are mostly reported to have an allelopathic interaction function, which can regulate the growth and development of themselves and the encompassing plant [24]. However, these compounds are widespread in many plants and have similar functions. As this review mainly aims to explore the characteristics of tea science, we have only focused on the specific accumulated metabolites in tea. The selected metabolites are either qualitatively or quantitatively different from other plant species. For example, l-theanine specifically and highly accumulates in tea plants, as reflected in the quality difference. Although catechins, caffeine, and volatile compounds are widely distributed in other plants, they are more abundant in tea plants, as reflected in the quantity difference. As these metabolites show specific accumulation in tea plants (Fig. 1A), research on their biological functions also elucidates their characteristics.

Many secondary metabolites are thought to be produced for resistance against biotic or abiotic stresses in plants [25]. Indeed, in some cases, metabolites are produced to help plants adapt to variable environments and grow normally. Therefore, whether abundant specialized metabolites have biological functions in tea plants is of interest. Compared with the studies on improving tea quality with stress, research on biological functions of metabolites in tea plants is limited. Most research has shown that many secondary metabolites in tea plants can respond to stress. However, the response does not represent the acting biological function. By surveying existing literature, we hope to evaluate the role of metabolites in tea plant growth comprehensively and objectively.

## Biological function of catechins in tea plants

As a major component of polyphenols, catechins account for 12–24% of dry tea [26]. Tea catechins comprise catechin (C), epicatechin (EC), gallocatechin (GC), epicatechin-3-gallate (ECG), epigallocatechin (EGC), and epigallocatechin-3-gallate (EGCG) [27]. C, EC, GC, and EGC are nongalloylated catechins, while ECG and EGCG are galloylated catechins. Abundant studies have shown that catechins have multiple effects on human health, playing an important role in antimicrobial, antiviral, and antiaging activity [28]. Many studies have explored the biological function of catechins in tea plants, achieving some progress.

As catechins play an important antimicrobial role in animals, they are also speculated to defend against diseases in tea plants (Table 2). Based on the differential accumulation of catechins in tea cultivars with different resistance abilities or catechin responses to abiotic stress, many studies have proposed that catechins might show resistance against stress. Blister blight is a disease caused by *Exobasidium vexans* in tea plants [29]. A positive correlation exists between EC content and resistance to *Exobasidium vexans*, suggesting that EC might play an important role in resistance to blister blight (Table 2) [29]. Another study also found a positive correlation between the EGCG and C contents in tea plants and resistance to *Colletotrichum fructicola*, and proposed that catechins might play a role in this resistance [8]. The functions and structures of catechins are quite different owing to the number of hydroxyl groups in the B-ring and presence or absence of a galloyl moiety [30]. Furthermore, the resistance of catechins containing gallate side chains, such as ECG and EGCG, is much higher than that of catechins lacking gallate side chains [31]. This suggests that the resistance of catechins to pathogens is mainly due to the presence of gallic acid (GA), which has strong antimicrobial activity [32]. In tea plants, *Exobasidium vexans* infection leads to a decrease in catechins (Fig. 1B) [33]. During infection, whether catechins undergo hydrolysis, and GA produced from catechin hydrolysis acts as a defense against pathogens, remains speculated, with further evidence needed for confirmation. There are few studies on the antipathogenic effect of catechins in tea plants. Most pathogens used in resistance evaluation experiments are unlikely to infect tea plants and might not cause the main diseases. Furthermore, the anti-infective activity differs significantly among different studies, probably owing to different *in vitro* experimental systems. Therefore, whether catechins have antipathogenic effects in tea plants and whether their content is sufficient to resist pathogens requires further study. In addition to responding to pathogens, catechins respond to insects. Transcriptome analysis showed that key synthetic genes of catechins were significantly upregulated by *Ectropis oblique* (Table 2) [9]. However, the catechin content in tea plants decreased after attack by *Helopeltis theivora* [34]. These studies suggested that catechins respond differently to different insects (Fig. 1B). However, according to current studies, the role of catechins against insects is unclear (Table 2; Fig. 1C).

**Table 2:** Potential biological function of specialized metabolites in tea plants.

Specialized metabolite	Potential biological function	Source of evidence	Reference
Catechin	Resistance to the infection of *Exobasidium vexans*	Correlation analysis between the content and resistance ability, content analysis	[29], [33]
	Resistance to the infection of *Colletotrichum fructicola*	Correlation analysis between the content and resistance ability	[8]
	Resistance to the attack of *Ectropis oblique*	Transcriptome analysis	[9]
	Resistance to the attack of *Helopeltis theivora*	Content analysis	[34]
	Defense against UV-B radiation	Content analysis	[36]
	Adapt to drought stress	Content analysis	[33]
l-Theanine	Nitrogen storage and transportation	Referred to the function of other nonprotein amino acids	[41]
	Detoxification of ammonia	Content analysis	[42]
	Precursor of specialized metabolite	Isotope tracing	[44], [45]
	Adapt to dark condition	Content analysis	[47]
	Adapt to salt stress	Content analysis	[50]
Caffeine	Resistance to the attack of *Ectropis oblique*	Transcriptome analysis	[9]
	Resistance to the attack of *Xyleborus fornicatus*	*In vitro* evaluation	[63]
	Resistance to the infection of *Colletotrichum fructicola*	Content analysis, *in vitro* evaluation	[8]
	Resistance to the infection of *Colletotrichum gloeosporioides*	*In vitro* evaluation	[65]
Volatile compound	Resistance to the attack of *Ectropis oblique*	*In vivo* evaluation	[75], [79]
	Resistance to the attack of tea green leafhopper	*In vivo* evaluation	[78]
	Adapt to low temperature stress	*In vivo* evaluation	[84], [85]

In addition to biotic stress, abiotic stress can also affect metabolite formation. To date, most reports on abiotic stress have focused on the effect of regulating light factors and drought on catechin metabolism. Light effectively activates the biosynthesis of catechins, resulting in higher contents than under dark conditions [35]. The effect of light on the synthesis of nongalloylated catechins was greater than that on the synthesis of galloylated catechins [27]. Furthermore, prolonged exposure to low UV-B radiation promotes catechin biosynthesis in tea plants [36]. These studies have shown that light intensity and quality regulate catechin biosynthesis (Fig. 1B), but the regulation mechanism involved has not been reported. Many studies have also investigated the effects of drought stress on catechin biosynthesis. At the start of drought stress, the catechin content increases (Fig. 1B), but then decreases with an increasing degree of drought [33]. However, most studies have focused on changes in catechins in response to stress (Table 2), with the related response mechanism seldom studied. In-depth research on catechin regulation under abiotic stresses not only aids understanding of whether catechins have biological functions to resist abiotic stresses, but also provides information for application of an external environment to regulate catechin biosynthesis.

## Biological function of l-theanine in tea plants

l-Theanine is a nonprotein amino acid found in tea plants, accounting for 1–2% of dry tea [27], [37]. Abundant nonprotein amino acids in plants have roles in nitrogen storage and transportation, defense against stress, and growth regulation [38]. l-Theanine not only affects tea quality, but also plays an important role in regulating tea plant growth. The main biological function of l-theanine is generally considered to be nitrogen storage and transportation (Table 2; Fig. 1C). In tea cultivation, nitrogen sources are generally in the form of ammonia (NH_4_-N) and nitrate (NO_3_-N). Compared with NO_3_^−^, tea plants prefer NH_4_^+^ as an inorganic nitrogen source, showing a higher absorption efficiency for NH_4_^+^ [39]. Excess ammonium is toxic to many plants, and millimolar concentrations of NH_4_^+^ can cause symptoms such as chlorosis or growth inhibition [40]. As an ammonia-tolerant economic crop, tea plants have different nitrogen metabolic pathways from other plants. l-Theanine plays an important role in this specific nitrogen metabolism pathway and is considered a nitrogen pool in nitrogen metabolism [41]. In tea plants, excess NH_4_^+^ absorbed by roots is converted into nontoxic l-theanine, which is transported to other tissues and then converted into other nitrogenous compounds. l-Theanine accumulation in tea plants increases with increasing NH_4_-N, with l-theanine considered to be a detoxification product of ammonia (Table 2) [42]. l-Theanine was proposed to be more easily transported in tea plants than l-glutamine, which has a similar structure [43]. In tea plants, l-theanine mostly synthesized in roots is usually transported to buds and converted into new compounds, such as other amino acids, catechins, and volatile compounds (Table 2) [44], [45]. Ethylamine hydrolyzed from l-theanine can be recycled into the catechin biosynthesis [44]. However, evidence for this conversion is currently based on isotope tracing experiments. Meanwhile, how ethylamine is used to synthesize catechins, and the reaction steps involved, remain unclear [46].

Tea plants contain l-glutamine, which has a greater nitrogen storage capacity than l-theanine. Therefore, the reason that l-theanine, which is formed *via* potentially toxic substance ethylamine, must be determined. A potential explanation is that l-theanine might play a more important role in tea plants, such as in resisting stress. Indeed, l-theanine might also play a role in resistance to stress, especially abiotic stress. l-Theanine metabolism changes under many abiotic stresses, such as light and salt stress. Metabolomics analysis has shown that light intensity affects the l-theanine content in tea plants, while shading treatment increases free amino acids, including l-theanine [47]. The activation of l-theanine synthesis in the shoots and roots of tea seedlings is related to shading treatment, with the conversion activity of ethylamine into l-theanine inhibited up to 50% by light [48], [49]. The results showed that the effect of light on l-theanine accumulation was related to the decrease in l-theanine synthesis (Fig. 1B). Another study has shown that salt stress can induce the upregulation of l-theanine synthesis (Fig. 1B) [50], suggesting that l-theanine might be similar to proline and glycine betaine, acting as an osmotic regulator under salt stress. Presently, research on l-theanine defense against abiotic stresses has only focused on the stress response, while the biological functions and potential mechanisms have not been explored or elucidated.

## Biological function of caffeine in tea plants

With multiple biological functions, caffeine plays an important role in the vegetative and reproductive growth of plants, such as protecting vegetative organs from harmful insects and pathogens, and attracting beneficial pollinators for their reproductive organs. Caffeine is widely recognized as a chemical that protects plants from damage, especially providing resistance against biotic stresses. In this chemical defense theory, the physiological function of caffeine forms a chemical defense system against herbivores and pathogens, as confirmed in many transgenic plants [51], [52], [53], [54], [55]. For example, transgenic tobacco and chrysanthemum with overexpression of genes involved in caffeine synthesis are apparently resistant to herbivores, such that caffeine is considered a natural insecticide [54], [55]. Furthermore, transgenic tobacco with abundant caffeine is resistant to pathogens, suggesting that plants can rely on the toxicity of caffeine to fight pathogens [56]. Owing to its bitter taste, caffeine inhibits insect feeding [54], [55]. Furthermore, caffeine acts as a repellent and toxicant against organisms primarily owing to the inhibition of phosphodiesterase activity and an increase in intracellular cyclic adenosine monophosphate [53]. In response to abiotic stresses, caffeine might also activate endogenous defense mechanisms by enhancing the salicylic acid (SA) content or defense-related gene expression [56], [57].

At the end of last century, caffeine had been found in 100 plant species, with the highest content in young leaves of *Camellia* (2–5% in *C. sinensis*) [58], [59]. In other species, the content is generally lower; for example, 2.5% in leaves of *Theobroma cacao* [60], 0.4–2.4% in seeds of *Coffea* [58], and less than 1% in young leaves of *Ilex paraguariensis* and flowers of *Citrus maxima* [61], [62]. Research has shown that the high caffeine content in tea plants might also play an important biological role, especially in anti-insect and antimicrobial responses. Transcriptome analysis has shown that *tea caffeine synthetase* (*CsTCS*) is upregulated in response to *Ectropis oblique* attack (Table 2) [9]. *S-Adenosylmethionine synthetase* (*CsSAMS*) and *CsTCS1*, key genes for caffeine synthesis, might be induced by *Colletotrichum fructicola* infection [8]. Furthermore, *in vitro* evaluation of the resistance and effects of exogenous treatment on tea plants has also been conducted. In laboratory control experiments, caffeine was found to inhibit the oviposition of *Xyleborus fornicatus* in tea plants and protect tender tissues from insect larvae (Table 2) [63]. A study has also shown that the caffeine content increased in tea plant stems after beetle infestation to inhibit the growth of *Monacrosporium ambrosium*, which is considered a defense strategy in tea plants (Table 2) [64]. Caffeine may have resistance to anthracnose, with an *in vitro* antifungal experiment indicating that the inhibitory effect of caffeine on mycelial growth was stronger than that of polyphenols [8]. Furthermore, applying caffeine to tea leaves significantly decreased infection from *Colletotrichum gloeosporioides* and *Colletotrichum gloeosporioides*-induced necrotic lesions (Table 2) [65]. Presently, studies on the biological function of caffeine in tea plants have only investigated the response of caffeine under biotic stress, evaluated *in vitro* resistance, or conducted phenotypic analysis after exogenous application (Fig. 1B and 1C), while *in vivo* evidence regarding the resistance role of caffeine is lacking [8], [9], [65]. Perhaps tea resources low in caffeine could be used to explore the biological function of caffeine. However, owing to different genetic backgrounds, these tea varieties might not be suitable materials. The availability of transgenic tea plants that are decaffeinated will facilitate thorough evaluation of the chemical protective effects of caffeine.

Caffeine is considered a biological defense in tea plants, protecting tender tissues from insects. However, insect pests remain a serious problem in tea plants, with tea plantation data from China showing that insect pests cause 10–20% of annual loss in tea production [66]. This indicates that major insects in tea plants might have evolved an adaptive detoxification system toward leaves with high caffeine contents. A study has shown that globally ubiquitous members of gut microbiota, including prominent *Pseudomonas* species, in insects subsist on caffeine as a sole source of carbon and nitrogen [67]. *Pseudomonas* caffeine demethylase reinstates the caffeine-degradation ability, indicating its key role in detoxifying caffeine in the gut microbiota of insects [67]. This study suggested that gut microbiota of major pests in tea plants might also have a caffeine detoxification function, but further exploration and verification is needed.

## Biological function of volatile compounds in tea plants

In response to environmental stress, plants release a mixture of volatile compounds for defense [68]. Compared with other secondary metabolites in tea plants, more research exists regarding the biological function of tea volatiles, especially their protective effects against herbivores (biotic stress). In nature, when attacked by herbivores, plants can release special chemical signals, known as herbivore-induced plant volatiles (HIPVs). HIPVs have direct and indirect defensive functions against herbivores, with indirect functions including attracting herbivore enemies and signaling within or between plants [68], [69]. In tea plants, research on HIPV resistance against herbivores has mainly focused on indirect defense, with some progress achieved in the last 30 years. As early as the 1990 s, with the development of analytical techniques, the role of HIPVs in mediating tritrophic plant–herbivore–carnivore interactions in tea plants was gradually revealed; for example, tea plant–*Toxoptera aurantii*–*Aphidius* sp./*Chrysopa sinica*/*Coccinella septempunctata* [70], and tea plant–*Tetranychus kanzawai*–*Neoseiulus womersleyi* [71]. In most studies, the attraction behavior of natural enemies toward volatile compounds is measured using an electroantennogram and Y-tube olfactometer. However, the response of natural enemies does not account for the defensive function of these signals. Furthermore, behavioral analysis of natural enemies using a Y-tube olfactometer does not reflect real conditions in a tea garden. Therefore, although studying volatile altruism has inspired the use of this indirect resistance mechanism to improve the efficiency of biological control, volatile altruism has yet to be applied in tea gardens.

**Fig. 2:**
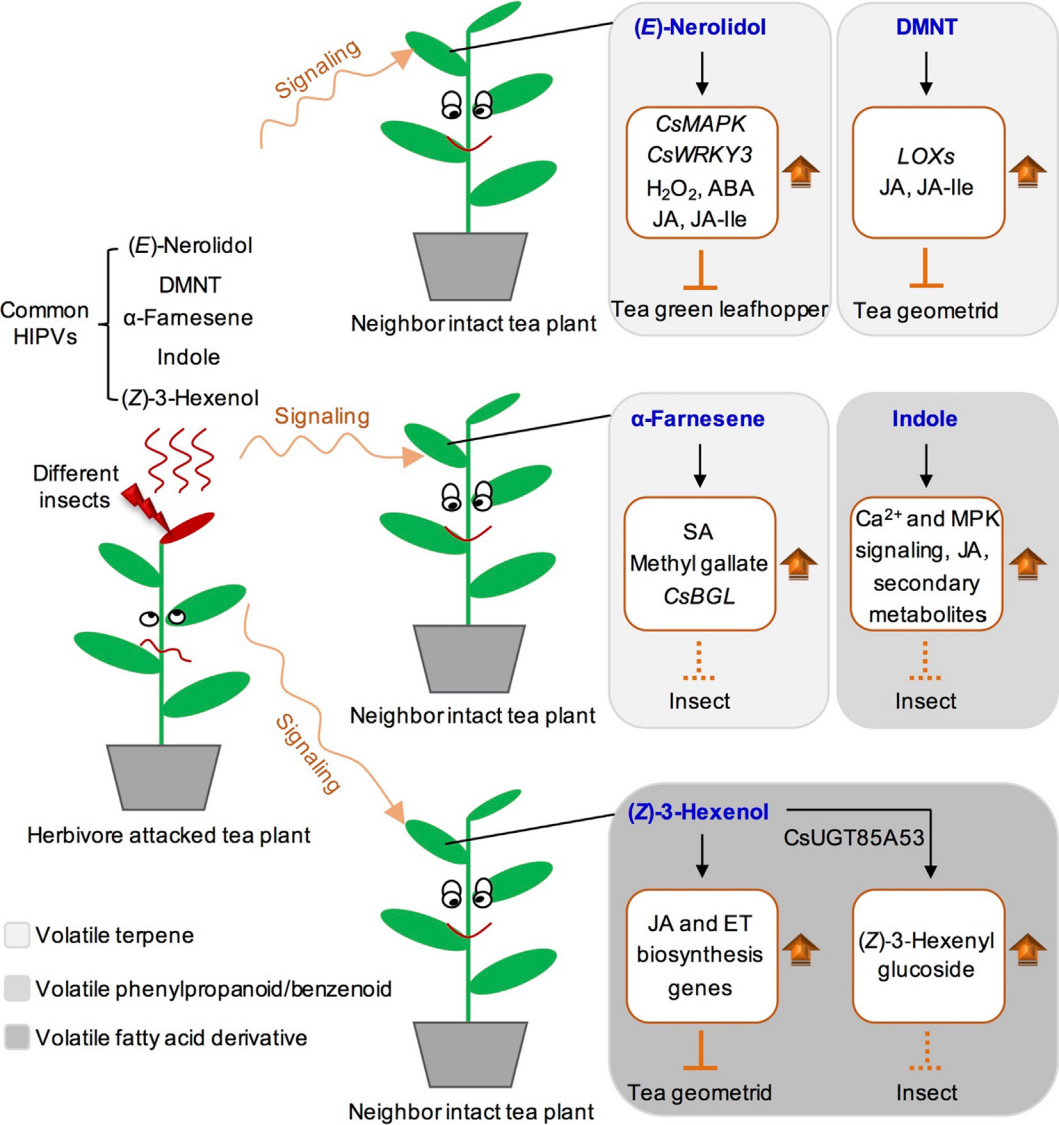
Plant-to-plant signaling of volatile compounds in tea plants. Abbreviation: ABA, abscisic acid; *BGL*, *β-1,3-glucanase*; CsUGT85A53, a glucosyltransferase; DMNT, (*E*)-4,8-dimethyl-1,3,7-nonatriene; ET, ethylene; JA, jasmonic acid; *MAPK* or MPK, *mitogen-activated protein kinase*; SA, salicylic acid. Solid line represents that the content has been confirmed by related studies, while dotted line represents that the content needs further evidence to confirm.

Research on HIPVs involved in signaling within or between plants has also made significant progress. In tea plants, HIPVs affect nonvolatile metabolite profiles in neighboring intact plants, and external signaling *via* HIPV might lead to more drastic changes in metabolite profiles of leaves than internal signaling *via* vascular connections [72], [73]. HIPVs released by different insects in tea plants have been investigated, showing that (*Z*)-3-hexenol, indole, (*E*)-nerolidol, (*E*)-4,8-dimethyl-1,3,7-nonatriene (DMNT), α-farnesene, and (*E*)-β-ocimene are common HIPVs (Fig. 1B) [72], [74]. Among them, (*Z*)-3-hexenol, an important green leaf volatile, activates defense against *Ectropis oblique* in tea plants [75]. The application of (*Z*)-3-hexenol in tea plants can significantly reduce the weight and increase the mortality of *Ectropis oblique* larvae, and promote the expression of genes related to JA and ethylene synthesis in intact plants (Fig. 2) [75]. Many plants have been confirmed to absorb (*Z*)-3-hexenol in air, which is converted into (*Z*)-3-hexenol glycoside to play an anti-insect role [76]. Recently, a study identified an important glycosyltransferase (GT, UGT85A53) related to glycosylation in tea plants, and elucidated the molecular mechanism of uridine diphosphate (UDP)-mediated glycosylation of (*Z*)-3-hexenol (Fig. 2) [77]. The expression of *mitogen-activated protein kinase* (*CsMAPK*) and *CsWRKY3*, which are stress response genes, was induced by exogenous (*E*)-nerolidol, and the contents of H_2_O_2_, abscisic acid (ABA), jasmonic acid (JA), and JA-Ile were also increased [78]. Furthermore, treated tea leaves showed a negative effect on the food intake of tea green leafhopper, demonstrating the resistance ability of (*E*)-nerolidol against tea green leafhopper (Fig. 2) [78]. DMNT, another volatile compound derived from (*E*)-nerolidol, also play an important role in the induction of JA-dependent herbivore resistance of neighboring tea plants through activating the expression of *lipoxygenases* (*CsLOXs*) (Fig. 2) [79]. In tea plants, α-farnesene might act as an interplant signaling molecule to enhance the resistance of neighboring intact tea plants by enhancing the contents of hormones (SA) and metabolites (methyl gallate), and the expression of related defensive genes (β*-1,3-glucanase*, *CsBGL*) (Fig. 2) [73], [80]. Although the insect-resistance properties of (*E*)-β-ocimene in tea plants have not been studied directly, their defensive effects have been confirmed in other plants. For example, in cabbage, exogenous fumigation with (*E*)-β-ocimene negatively affects aphid feeding behavior [81]. Indole also acts as a priming signal between neighboring plants to defend against insects [82]. Recently, a study extends the molecular basis of indole-induced defense priming in tea plants. It found that that exposure to indole primes the expression of early defense genes involved in calcium (Ca^2+^) and MPK signaling, and the biosynthesis of JA and defense-related secondary metabolites (Fig. 2) [83]. In a word, common HIPVs can resist different insects in tea plants and other plants, and might be broad-spectrum resistant candidates to participate in defense against multiple insects.

Recently, volatile compounds have been reported to enhance the low-temperature resistance of tea plants through signal transduction. A study has provided the first evidence of interplant communication *via* inducible volatile compounds under cold stress [84]. This indicates that many volatile compounds, including (*E*)-nerolidol, geraniol, linalool, and methyl salicylate, emitted from cold-stressed tea plants play key roles in priming the cold tolerance of neighboring plants *via* a C-repeat binding factor (CBF)-dependent pathway (Table 2; Fig. 1B and 1C). Furthermore, exogenous fumigated (*E*)-nerolidol can be converted into glycosylated nerolidol by GT, and glycosylated nerolidol can enhance the scavenging of reactive oxygen species in tea plants, which improves cold resistance [85]. This study also identified that *CsGT* (*UGT91Q2*) controls (*E*)-nerolidol glycosylation in plants and a new function of (*E*)-nerolidol in regulating cold resistance in tea plants [85]. Compared with studies on insect resistance, studies on volatile resistance to abiotic stress are limited.

(*Z*)-3-Hexenol is widely recognized as a defense substance against insects in tea plants and other crops [75], [76]. However, this substance has also been reported as an attractant for tea green leafhoppers [86], [87]. Therefore, the real function of (*Z*)-3-hexenol in tea plants, and why it has two opposing roles of, defense and attraction, requires elucidation. Several studies have shown that plant volatiles play an important role in regulating the dynamics of pest communities, but their application in actual tea gardens has yet to be reported. Presently, most evaluations of insect resistance have been performed under controlled conditions. However, the factors involved in tea gardens are much more complex, potentially causing differences between results from laboratory control experiments and field experiments. Many volatile compounds have anti-insect functions in laboratory control experiments, but the function is obviously weakened in the field environment. This phenomenon raises the question of whether the ecological functions of these volatiles are actually present in tea gardens. Current evidence might not be sufficient to answer this question. Adjustments might be needed to the current evaluation system, or even to the experimental design. The discovery of insect-resistant phenotypes should be conducted in the field and then mechanism elucidation should be conducted in the laboratory. Volatile components from tea resources with different insect-resistances and degrees of insect pests in the field have been investigated to obtain possible insect-resistant candidates from field data. Functional evaluation of candidates in the field and a study on the potential resistance mechanism in the laboratory were conducted. The combined effect of volatile compounds with different compositions or proportions should also be considered in future. In tea gardens, the role of volatile substances is affected or weakened by many external factors, such as wind and distance. These experiments will present further difficulties that need to be overcome. However, insect-resistant substances obtained using this strategy might play a real defensive role in tea gardens. Related studies are beneficial for the discovery and application of environmentally friendly chemical pheromones in tea gardens, which will improve tea quality more safely.

## Environmental adaptability of tea plants

The normal growth of any plant cannot be separated from a certain suitable environment and its adaptability to the environment. Tea plants have evolved a set of systems to adapt to environments during long-term growth and reproduction. Researchers have attempted to elucidate the mechanism of adaptability of tea plants to the environment. According to current popular topics and research progress, we propose that the main characteristic scientific questions regarding tea plant growth in contrast to other economic crops are: (i) How tea plants adapt to acid soils; (ii) why tea plants have fewer diseases; and (iii) why tea plants and tea green leafhoppers have a symbiotic relationship. Accordingly, we have summarized the potential adaptive mechanism, and proposed future research that needs to be conducted.

## How tea plants adapt to acid soils

**Fig. 3:**
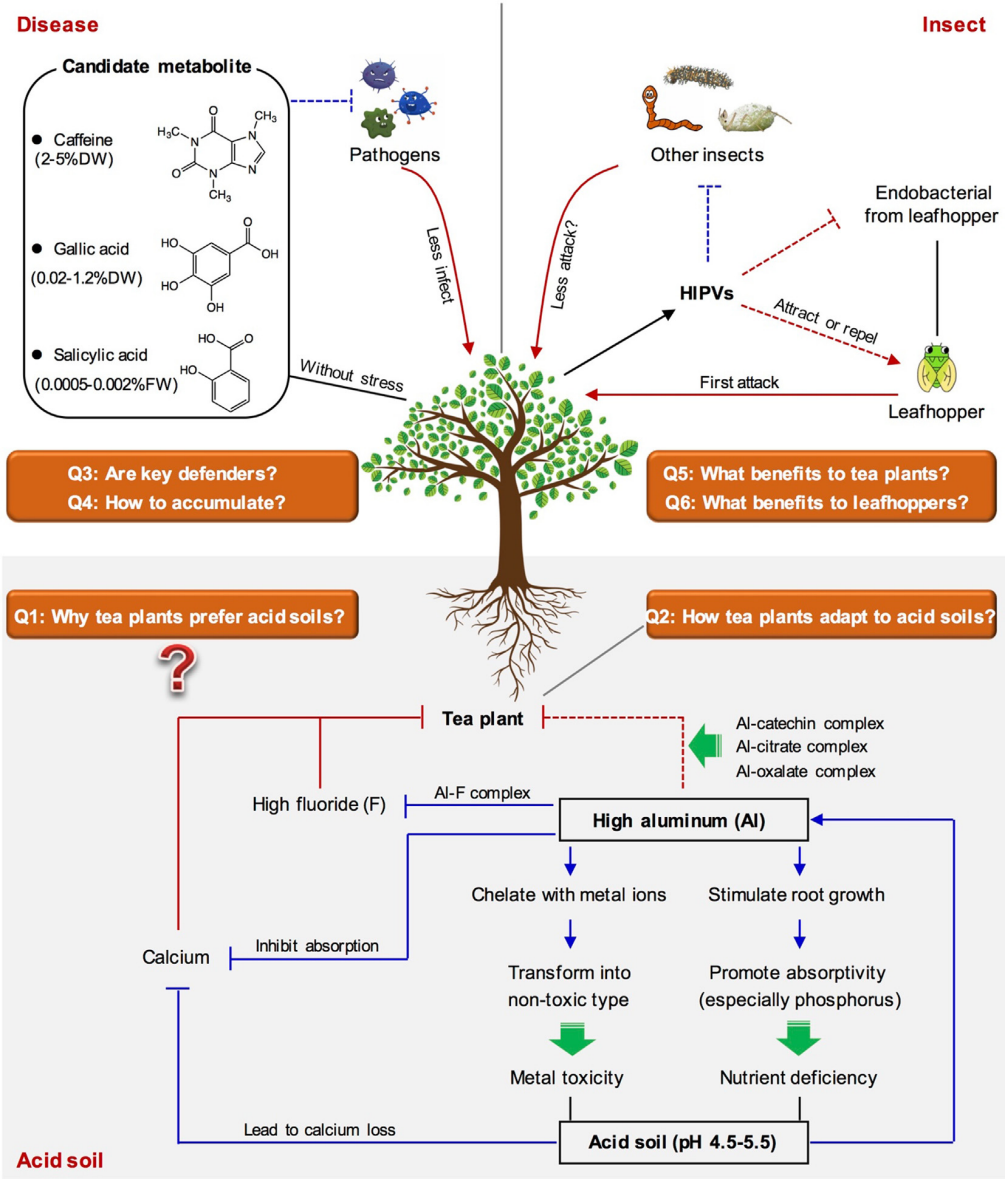
Proposed mechanism involved in adaptability of tea plants to environment. Abbreviation: HIPVs, herbivore-induced plant volatiles; Al, aluminium. Q1-6, main questions involved in adaptability of tea plant to environment. Solid line represents that the content has been confirmed by related studies, while dotted line represents that the content needs further evidence to confirm.

Although soils that are too acidic are not suitable for most plants, tea plants can grow in acid soils and grow well at pH 4.5–5.5 [88], [89]. Tea plants have long been known to favor acid soils, but the reason remains unclear. Understanding why tea plants prefer acid soils (Fig. 3, Q1) is useful for elucidating the effects of soil environment on tea quality and active components, and also determining whether planting in acid soils is actively demanded or passively selected. Acid soils can cause many problems, such as metal toxicity, nutrient deficiency, and inhibition of soil microbial growth [90]. However, tea plants grow well in acid soils, indicating that they are highly acid-tolerant. In contrast, considerable research has been conducted on how tea plants adapt to acid soils (Fig. 3, Q2). Why tea plants need to be grown in acid soils and how to adapt to acid soils are two different scientific questions. To date, most studies have focused on the adaptation to acid soils, with no clear explanation provided for why acid soils are needed.

Aluminum (Al) is the main toxic metal restricting plant growth in acid soils [91]. Al is the most abundant metal element in soil, generally existing as Al^3+^ in acid soils, with the Al^3+^ concentration increasing with increasing acidity [92]. In general, high-concentration soluble Al^3+^ is not conducive to the growth of many plants, mainly affecting their root development [93]. However, as an Al hyperaccumulator, tea plants do not exhibit any toxic symptoms with a high-concentration Al supply, while the absorption of large amounts of Al from the soil is beneficial to its root growth [94], [95], [96]. Al has also been shown to affect the activity of genes and enzymes controlling the synthesis of pectin and hemicellulose, which can loosen cell walls and promote tea root elongation [95]. Furthermore, a recent study has provided novel evidence that Al^3+^ helps to maintain DNA integrity in meristematic cells to promote root growth in tea plants [96]. In tea plants, Al mainly accumulates in the cell wall of mature leaves and is chelated with catechin (Fig. 3) [97], [98]. *In vitro* experiments have shown that catechins competitively occupy the coordination sites of Al [99] and accumulate abundantly in leaves. This might explain why Al in tea plants mostly exists in the form of Al–catechin complex. Furthermore, a recent study proved that Al exists as an Al-EGCG complex in tea plants through liquid and solid ^27^Al nuclear magnetic resonance [100]. The authors also confirmed that Al can form a complex with proanthocyanidin in the root. Although the presence of Al can disrupt the growth and development of many plants, some other plants, such as tea and coffee plants, require Al to promote their growth, suggesting that Al has special biological functions. For example, fluoride (F), another highly accumulated element in tea plants, is destructive to tea plants in the absence of metal, but formation of an Al–F complex when Al is present can aid detoxification (Fig. 3) [101]. Further exploration of the biological function of Al can aid understanding of the demand for Al and acid soils in tea plants. In addition to Al, acid soils activate other toxic metal ions that are harmful to tea plant growth. In tea plants, Al also plays an important role in the resistance to toxicity caused by other elements, particularly zinc (Zn), manganese (Mn), and copper (Cu) [102]. Furthermore, organic acids secreted by the root are also related to the detoxification of heavy metals in plants [103]. Many complexes of Al with organic acids, such as Al–citrate and Al–oxalate complexes (Fig. 3), have also been found in the xylems and root sap of tea plants [104], [105]. However, the contribution of these organic acids to the detoxification of Al in tea plants and the specific mechanism of detoxification are yet to be determined.

Nutrient deficiency is another important factor limiting normal plant growth in acid soils. Phosphorus (P) is an essential nutrient for plant growth and development, and acid soils are seriously deficient in P. However, when tea plants grow in acid soils, the presence of Al stimulates root growth, which increases the root area to promote P absorptivity (Fig. 3) [94], [106]. Furthermore, acid soils readily cause the loss of many metal elements, especially calcium (Ca), which affects plant growth and development [107]. The high Al concentration in acid soils also competitively inhibits Ca uptake (Fig. 3) [108]. As a calcifuge plant, the demand for Ca in tea plants is much lower than in most plants, with too much Ca affecting their normal growth [109]. Therefore, the high Al content and Ca loss from acid soils might be intended to provide low-Ca conditions for tea plants. However, the cause of the calcifuge property of tea plants remains unclear and requires further research. The soil environment of tea plants contains numerous microbes, including arbuscular mycorrhizal fungi, which will greatly improve the nutrient absorption and alleviate nutrient deficiency induced by acid soil [110]. Furthermore, soils that are too acidic generally negatively affect the survival of soil microorganisms, destroying plant development. However, in tea garden soils, the main microbes are Al-tolerant fungi [111], which might result from coevolution and help tea plants adapt to acid soils.

Acid soils can effectively address the adverse effects of F and Ca on tea plants. Therefore, the acid soil might be intended to alleviate these effects and provide a high-Al environment conducive to tea plant growth. As an Al hyperaccumulator, tea plants can grow well under acid conditions, and are among the best materials for studying the physiological mechanisms of Al tolerance and acid stress. The high accumulation of Al in tea plants indicates that Al might play a unique biological role in plant growth. However, current research on Al in plants mostly focuses on Al toxicity to plants and the detoxification mechanism of plants, with the biological function of Al seldom studied. Research on the biological function of Al will help elucidate why tea plants require so much Al, and might explain why they require acid soils. Today, with increasing soil acidification, declining soil fertility and toxic metal activation affect the growth of many crops. Therefore, elucidating the acid tolerance mechanism of tea plants is important for promoting the adaption of crops to the environment and developing green agriculture.

## Why tea plants have fewer diseases

As a perennial woody plant, tea plants are subjected to various biotic and abiotic stresses during their long-term growth. Disease is a common biotic stress that affects many other economic plants, leading to a significant reduction in yield [112]. Tea plants are mostly grown in warm and humid tropical and subtropical regions suitable for the reproduction of various pathogens. Indeed, many types of pathogen have been found in tea plants; for example, more than 100 microbial diseases have been found in tea gardens in China [113]. However, compared with other economic plants, the harm caused by diseases in tea plants are not serious, rarely causing huge economic losses. This is probably attributed to the evolved defense systems of tea plants, and the developed physiological adaptations enhancing their resistance to pathogen infections.

Tea plants are rich in secondary metabolites, even under stress-free conditions. In combination with analysis of the secondary metabolites enriched in tea and their biological functions, we suggest that three metabolites, namely caffeine, SA, and GA, might be the key candidates helping tea plants to resist diseases (Fig. 3). As discussed above, caffeine is proposed to have an important disease-resistance function in plants [56], [64]. Similarly, caffeine abundantly accumulates in tea plants [58], [59] and is reported to be involved in the defense against main pathogens of tea plants [8]. Therefore, this metabolite is considered a key candidate against tea diseases. SA acts as an endogenous signal mediating systemic and local defense against pathogens in plants [114]. In many plants, such as *Arabidopsis thaliana*, *Nicotiana tabacum*, *Cucumis sativus*, and *Capsicum annuum*, SA levels are generally low without stress, ranging from 0.01 to 0.05 μg/g (FW), while clearly increasing when infected by pathogens, but remaining under 5 μg/g (FW) [115]. However, the level of SA in tea plants is high, at 5–20 μg/g (0.005–0.002% FW) without stress [10], [22], [78], [116]. Therefore, the high accumulation of SA in tea plants is speculated to contribute to resistance against many diseases. In addition to these metabolites, GA might also play an important role in disease defense in tea plants. GA not only accumulates abundantly (0.2–12 mg/g, 0.02–1.2% DW) in tea plants, but also acts as the skeleton structure of galloylated catechins [11], [117], [118]. Many studies have shown that GA has strong antibacterial effects [32], [119]. However, most studies on the antimicrobial activities of these metabolites in tea plants focus on the response to biotic stress and resistance evaluation *in vitro* (Fig. 3, Q3). Furthermore, the reason for the abundant accumulation of these metabolites in tea plants is worth further exploration (Fig. 3, Q4) to provide guidance for cultivating high-resistance resources.

Plant defense includes constitutive traits and inducible responses [25]. In addition to the above constitutive metabolites, tea might have many inducible metabolites participating in disease resistance, such as volatile metabolites [78], [120], [121]. In fact, as a complex individual, the tea plant is rich in various secondary metabolites, whose *in vivo* functions may differ from evidence *in vitro*. To elucidate their biological functions, individual plant–pathogen combinations must be studied on a case-by-case basis, accounting for both host and pathogen responses. In-depth study of the biological functions of tea secondary metabolites will provide further ideas for plant resistance research. Biological control can perhaps replace chemical fungicides in future, reducing pollution and protecting the environment.

Endophytes have also been reported to play an important role in biotic stresses, such as disease defense, in many plants [122]. They directly antagonize pathogen growth by competing for nutrition and space, or indirectly inhibit pathogen growth by producing secondary metabolites with antibacterial activity [123], [124]. Similarly, a large number of endophytes have been identified and isolated in tea plants [125], [126]. A recent review provided a good overview of the distribution characteristics, diversity, and biological functions of tea plant endophytes [127]. The strain isolated from *C. sinensis* cv. Tieguanyin leaves, *Bacillus subtilis* TL2, has been proven to inhibit the growth of four pathogenic bacteria in tea through *in vitro* experiments [128]. Furthermore, the cell-free culture filtrate of the *Colletotrichum gloeosporioides* CgloTINO1 strain isolated from Indian tea gardens can inhibit the growth of tea pathogens [129]. This shows that the endophytes of tea plants might participate in resisting stress from pathogenic bacteria directly or indirectly. Different seasons, planting environments, and tea plant species, among other factors, will affect the types of endophytes in tea plants [125], [127]. Therefore, the dominant microorganism in different tea plants are different, and current research on disease resistance by tea endophytes mostly uses *in vitro* experiments and lacks *in vivo* evidence. Accordingly, more research is needed to provide more direct evidence.

## Why tea plants and tea green leafhoppers have a symbiotic relationship

During tea plant growth, many pest insects including tea green leafhoppers, tea geometrids, and tea aphids, attack leaves, reducing the tea yield and quality [72]. Among them, tea green leafhoppers are widely distributed in tea producing areas, and are among the most harmful pests in tea production. Tea green leafhoppers are more closely related to tea plants than other pests, and are found wherever tea plants are present. A symbiotic relationship might exist between tea plants and tea green leafhoppers, both of which have evolved to adapt to each other. In tea plants, attack by tea green leafhoppers induces the formation of HIPVs [72]. Many studies have proposed that plant-generated HIPVs play a defensive role against insects [68], [69]. Although only a few reports on HIPV defense against tea green leafhopper have been published to date [78], they confirm the existence of this function in tea plants. Some HIPVs are good for tea plants, while some are good for insects. For example, some volatile compounds, including (*E*)-2-hexenal, (*Z*)-3-hexenol, and (*Z*)-3-hexenyl acetate, are highly attractive to tea green leafhoppers when mixed in a certain proportions [87], with further study determining the proportion that is most attractive to tea green leafhoppers in tea gardens [86]. These studies have all shown that HIPVs induced by tea green leafhopper attack are attractive to the insect, which is beneficial to its feeding. Furthermore, HIPVs might influence insect feeding physiology by regulating microbes [130], [131]. Tea green leafhopper has also been found to induce geraniol release, but has no effect on the activity of geraniol synthase (GS) in tea leaves [132]. However, GS from tea green leafhopper can induce geraniol formation in tea plants, suggesting that the protein from this insect might be the main factor. Furthermore, geraniol inhibited the endobacteria isolated from tea green leafhoppers *in vitro*. Therefore, during attack, tea green leafhoppers are speculated to use their own GS to enhance geraniol formation in tea plants, with the produced geraniol then being able to affect the insect physiological activity by mediating the growth of its endobacteria [132].

Therefore, based on current research, we have developed hypotheses regarding the symbiotic mechanism of tea plants and tea green leafhoppers (Fig. 3). The formation of a symbiotic relationship mainly depends on HIPVs. Some HIPVs might protect tea plants by reducing tea green leafhopper invasion, while others might affect the physiological activity of tea green leafhoppers by attracting them or mediating endobacteria. These aspects promote a mutually beneficial relationship between the two organisms. Furthermore, whether the possible symbiotic relationship, or HIPVs mediating this relationship, can reduce tea plant attack by other insects is worth exploring, and will help explain the mutually beneficial relationship between tea plants and tea green leafhoppers at the insect community level. For tea plants, this mutually beneficial relationship reduces the damage from other insects, while reducing food competition for tea green leafhoppers. This hypothesis requires extensive evidence to be verified. Only a comprehensive understanding of the benefits to tea plants and tea green leafhoppers during attack might shed light on the symbiotic relationship between these two organisms (Fig. 3, Q5 and Q6).

## Concluding remarks and perspectives

This review summarizes important research progress regarding the biological function of specialized metabolites, including catechins, l-theanine, caffeine, and volatile compounds, in tea plants. Among these metabolites, investigation of the biological functions of volatile compounds in tea plants is the most advanced. Presently, some direct *in vivo* evidence of plant-to-plant signaling of volatile compounds in tea plants has been obtained, and the underlying mechanisms have been partly elucidated. Furthermore, the potential adaptive mechanism of tea plants to the environment has been summarized. Abundant specialized metabolites have been speculated to contribute to the adaptability of tea plants to the environment. This review provides many candidate target substances for green control of tea plants, and improves understanding of the growth mechanism of acidophilous plants, promoting progress in agricultural and biotechnological industries and helping establish green food in the food industry.

However, each aspect now requires further *in vivo* evidence and further exploration of relevant mechanisms. In addition, studying the biological function of characteristic tea plant metabolites and their roles in adaptation of the tea plant environment will help elucidate the origin, evolution, and spread of the tea plant. Related research also helps to provide a theoretical basis for improving tea cultivation measures and guides practical tea garden management. In-depth study of the biological functions of the secondary metabolites and environmental adaptation of tea plants, and the combination and application of laboratory and field experimental results, are needed. Presently, many laboratory experiments show significant effects. For example, many volatile compounds show significant insect-resistance effects *via* plant-to-pant signaling in tea plants. However, the effects of many main factors selected in laboratory experiments are obviously weakened during actual production in tea gardens owing to various complex factors. Therefore, research on biological function and environmental adaptation should be based on actual problems in tea gardens involving multiple factors, such as the influence of insects, pathogens, and climate. Furthermore, relevant experiments based on actual factors occurring in tea gardens must be designed and conducted to solve practical problems in the tea industry.

## Compliance with Ethics Requirements

*This article does not contain any studies with human or animal subjects*.

## Declaration of Competing Interest

*The authors declare that they have no known competing financial interests or personal relationships that could have appeared to influence the work reported in this paper*.
